# The role of LncRNA LBX2-AS1 in cancers: functions, mechanisms and potential clinical utility

**DOI:** 10.1007/s12094-022-02944-2

**Published:** 2022-09-21

**Authors:** Yuanshuai Su, Chengzhi Li, Yu Fang, Xinyu Gu, Qiuxian Zheng, Juan Lu, Lanjuan Li

**Affiliations:** 1 State Key Laboratory for Diagnosis and Treatment of Infectious Diseases, National Clinical Research Center for Infectious Diseases, National Medical Center for Infectious Diseases, Collaborative Innovation Center for Diagnosis and Treatment of Infectious Diseases, The First Affiliated Hospital, Zhejiang University School of Medicine, No. 79 Qingchun Road, Shangcheng District, Hangzhou, 310003 Zhejiang China; 2 Department of Pathology, The First Affiliated Hospital, Zhejiang University School of Medicine, Hangzhou, 310003 Zhejiang China

**Keywords:** Long noncoding RNA, LBX2-AS1, Oncogenic, Molecular mechanism, Cancer therapy

## Abstract

Increasingly advanced biology technique has revealed that long non-coding RNAs (lncRNA) as critical factors that exert significant regulatory effects on biological functions by modulating gene transcription, epigenetic modifications and protein translation. A newly emerging lncRNA, ladybird homeobox 2 (LBX2)-antisense RNA 1 (LBX2-AS1), was found to be highly expressed in various tumors. Moreover, it is functionally linked to the regulation of essential tumor-related biological processes, such as cell proliferation and apoptosis, through interactions with multiple signaling molecules/pathways. The important roles played by LBX2-AS1 in cancer initiation and progression suggest that this lncRNA has enormous clinical potential for use as a novel biomarker or therapeutic target. In this article, we retrospectively review the latest advances in research exploring the roles of the lncRNA LBX2-AS1 in oncology field, highlighting its involvement in a comprehensive network of molecular mechanisms underlying diverse cancers and examining its potential applications in clinical practice.

## Introduction

Cancer is a devastating disease that can be difficult to cure and occurs in various organs, involving multiple, complex biological factors [1]. Despite great advances in cancer therapy, cancer continues to be a challenging issue for global health systems, associated with a high incidence and high mortality rates [2, 3]. Additionally, traditional cancer intervention modalities, such as radiotherapy, surgery, and chemotherapy, can induce severe side effects in patients, with negative consequences on quality of life [4–6]. According to Global Cancer Statistics 2020, approximately 19.3 million new cancer cases are reported annually, in addition to nearly 10 million cancer-related deaths worldwide [2]. In recent decades, major breakthroughs in biotechnology have expanded the body of knowledge regarding the complex genomics and immune system functions underlying cancer development and progression, leading to the development of precise cancer therapies that target specific molecules involved in carcinogenesis [7–11]. To treat malignancies more precisely, further investigations into promising new biomarkers and therapeutic targets remain necessary.

Non-protein-coding sequences in the human genome were long regarded as useless, with no functional contributions to biological processes. However, in recent years, rapid developments in high-throughput sequencing have resulted in high-resolution and large-scale studies that have provided a new perspective on genetic materials that lack protein-coding capacity [12, 13]. Although protein products are encoded by only a small proportion of the genome, large quantities of nucleotides can be detected to transcript [14]. Multiple types of non-coding RNAs have been identified, including long non-coding RNAs (lncRNAs), which are defined as non-protein-coding transcripts containing more than 200 nucleotides [15]. The human genome is estimated to encode at least 20,000 lncRNAs, which can be divided into four primary functional categories: scaffold, decoy, guide, and signaling [1, 16–18]. The majority of lncRNAs are localized in the nucleus and are involved in various biological processes, such as gene expression and chromosomal remodeling, through interactions with key regulators [19, 20]. However, the biological functions and underlying mechanisms of diverse lncRNAs have not been described gorgeously. In the past few decades, accumulating studies have revealed that lncRNAs function to promote or suppress oncogenesis, exerting significant effects on cancer processes [21–24]. For example, the lncRNA prostate cancer gene expression marker 1 (PCGEM1) enhances tumor malignancy by regulating CDK6 expression [25]. LncRNA H19 is capable of impeding telomerase function by disrupting the hTERT (Human telomerase reverse transcriptase)-hTR (Human telomerase RNA) interaction, which could be targeted for therapeutic purpose [96]. Alain Chebly et al. demonstrated the participation of TERRA (telomeric repeat-containing RNA) in CTCL (cutaneous T-cell lymphomas) and the role as a modulator of canonical telomerase functions [97]. In-depth investigations have not only expanded our understanding of the relationships between lncRNAs and cancer biology but have also provided us with theoretical bases for the development of novel biomarkers and therapeutic targets.

Ladybird homeobox 2 (LBX2)-antisense RNA 1 (LBX2-AS1) is a recently discovered lncRNA that has been reported to play regulatory roles in diverse human cancers. LBX2-AS1 is encoded by a gene on chromosome 2p13.1 that lacks any protein-coding capability. Subcellular localization analysis revealed that LBX2-AS1 is primarily distributed to the cytoplasm, with a small proportion found in the nucleus [26–29]. Over the past two decades, accumulated studies have demonstrated that LBX2-AS1 exerts significant effects on the occurrence and development of various human cancers, such as ovarian cancer (OC), glioma, and gastric cancer (GC) [30–32]. LBX2-AS1 plays powerful regulatory functions in tumor progression as an oncogenic factor by interacting with diverse signaling molecules, impacting the biological characteristics of various cancer cells, including altering their invasive abilities, proliferation capacities, and drug resistance properties. LBX2-AS1 was also implicated to influence non-cancer diseases, such as periodontitis [33]. However, the underlying mechanisms through which LBX2-AS1 exerts tumor-related functions in diverse cancer types and the regulatory networks involved in these functions, including upstream elements and downstream pathways, have not yet been comprehensively elucidated. However, both in vitro studies and in vivo animal experimentation have demonstrated the tremendous clinical potential of LBX2-AS1.

In this article, we review recent research findings regarding the oncogenic roles played by the lncRNA LBX2-AS1, with a focus on LBX2-AS1 expression profiles in diverse cancer types and the relationships between LBX2-AS1 dysregulation and the clinical features of human cancers. Furthermore, the biological functions, complex molecular mechanisms, and promising clinical value of LBX2-AS1 are systematically discussed.

## Relationship between LBX2-AS1 expression and clinicopathological characteristics of diverse human cancers

Numerous studies have reported increased LBX2-AS1 expression in various human cancers, and associations between LBX2-AS1 expression levels and the clinical characteristics of tumors have also been described. In this section, we will discuss the dysregulated expression profiles of LBX2-AS1, with particular emphasis on relevant cancer-related clinical features and tumor growth traits in xenograft models (Table 1).

**Table 1:** Expression files of LBX2-AS1 and relevant clinicopathological characteristics

Cancer type	Expression	Samples	Animal experiment	Clinicopathological features	References
GC	Upregulated	40 GC tissues and paired adjacent non-tumor tissues	Tumor growth (growth rate, volume and weight), IHC staining	Survival rate	[30]
GC	Upregulated	–	Tumor growth (growth rate, volume and weight), H&E staining and IHC staining	–	[39]
GC	Upregulated	GC tissues and paired adjacent non-tumor tissues from 78 patients	–	–	[40]
OC	Upregulated	60 ovarian cancer tissues and paired adjacent non‐tumor tissues	Tumor growth (rate volume weight)	Survival rate	[47]
OC	Upregulated	Ovarian cancer tissues and paired adjacent non-tumor tissues from 24 patients	Tumor growth (rate volume weight)	–	[31]
CRC	Upregulated	256 CRC tissues and paired adjacent non-tumor tissues	Drug reaction	TNM stage, local invasion, lymph node metastasis, survival rate, 5-fluorouracil response	[44]
CRC	Upregulated	–	–	Survival rate	[29]
CRC	Upregulated	145 CRC tissues and paired adjacent non-tumor tissues	–	TNM stage, lymph node metastasis, survival rate	[26]
CRC	Upregulated	48 CRC tissues and paired adjacent non-tumor tissues	–	Tumor volume, metastasis	[104]
Glioma	Upregulated	30 Glioma tissues and 5 non-tumor tissues	Tumor growth (rate volume weight), IHC staining	Tumor subtype, tumor recurrence, survival rate	[32]
Glioma	Upregulated	–	–	Prognosis	[77]
HCC	Upregulated	HCC tissues and paired adjacent non-tumor tissues from 45 patients	Tumor growth (rate volume weight)	TNM stage, lymph node metastasis, survival rate	[52]
TC	Upregulated	510 TC tissues and paired adjacent non-tumor tissues	Tumor growth (rate volume weight), IHC staining	T, N Stage	[27]
MM	Upregulated	Tumor tissues and serum samples from 60 MM patients and healthy controls	Tumor growth (rate volume weight)	–	[28]
NSCLC	Upregulated	165 NSCLC tissues and paired adjacent non-tumor tissues	–	TNM stage, histological grade, lymph node metastasis, survival rate	[53]
ESCC	Upregulated	82 ESCC tissues and paired adjacent non-tumor tissues	–	Tumor metastasis	[54]

### Tissue samples and cell lines

#### Gastric cancer

According to Global Cancer Statistics 2020, over 1 million people were newly diagnosed with GC in 2020, and GC was responsible for approximately 769,000 deaths, making GC the fifth-most commonly diagnosed cancer type and the fourth leading cause of cancer-related death worldwide [2]. Due to non-specific symptoms and poor prognosis, GC remains a huge global health burden [34, 35]. Due to the limited availability of effective therapeutic targets and credible biomarkers capable of identifying early-stage GC, improvements of GC interventions remain urgently necessary [36–38]. Recent investigations have demonstrated that the aberrant expression of the lncRNA LBX2-AS1 is significantly correlated with GC initiation and progression. LBX2-AS1 is expressed at significantly increased levels in GC tissues compared with adjacent non-tumor tissues and is upregulated in GC cells compared with non-tumor-derived gastric mucosa epithelial cells (GES-1) [30, 39, 40]. Moreover, Kaplan–Meier analyses revealed that LBX2-AS1 expression levels were negatively associated with survival rates among patients with GC, suggesting that LBX2-AS1 may serve as a key prognostic indicator [30].

#### Colorectal cancer

With more than 1.9 million new cases and approximately 935,000 corresponding deaths in 2020, colorectal cancer (CRC) is the third-most commonly occurring cancer and the second leading cause of cancer-related death worldwide. Despite improved early diagnosis and treatment approaches, death rates continue to increase, particularly in rural areas and less technologically advanced countries that lack medical resources [41–43]. Recent studies demonstrated that LBX2-AS1 is critically upregulated in most CRC tissues compared with adjacent non-tumor tissues, and LBX2-AS1 expression levels gradually increase in parallel with advanced CRC tumor stages. Consistently, LBX2-AS1 expression is elevated in CRC cell lines compared with non-tumor cells [26, 29, 104, 106]. More importantly, analysis of CRC survival data revealed that LBX2-AS1 expression levels were negatively correlated with overall survival among patients with CRC [26, 29, 44]. However, no relationship was identified between disease-free survival and LBX2-AS1 expression [26, 44]. Further analysis indicated that high levels of LBX2-AS1 expression were significantly associated with advanced tumor–node–metastasis (TNM) staging, frequent local invasion, and extensive lymph node metastasis, which are aggressive characteristics of CRC. Clinical investigations of patients with CRC demonstrated that high expression level of LBX2-AS1 is correlated with 5-fluorouracil (FU) treatment resistance [44].

#### Ovarian cancer

OC is one of the most frequently diagnosed cancer types among women and is the deadliest gynecologic malignancy, accounting for 313,959 new cases and 207,252 cancer-related deaths in 2020 [2]. The 5-year survival rate for OC has remained at approximately 47%, with little improvement for nearly two decades. OC also has a high recurrence rate, making early detection and the development of effective treatment measures important research goals [45, 46]. In recent years, critically elevated LBX2-AS1 expression levels have been detected in OC tissues and OC cell lines compared with adjacent normal tissues and normal cell lines, respectively, indicating that LBX2-AS1 might be an oncogenic lncRNA [31, 47]. More remarkably, the overexpression of LBX2-AS1 was significantly correlated with shortened survival among patients with OC [47]. In another bioinformatics study, Meng et al. performed an overall analysis of autophagy-related lncRNAs associated with OC, and LBX2-AS1 was one of the 17 aberrantly expressed lncRNAs that were selected as components of an independent prognostic model [48].

#### Glioma

Glioma is the most frequent primary cancer of the central nervous system, accounting for approximately one-third of all neural malignancies. Despite its rare occurrence, glioma leads to prominent mortality [49, 50]. Recent studies revealed that in glioma tissue samples and cancer cell lines, LBX2-AS1 was detected to increase critically through qRT-PCR and FISH (Fluorescence in situ hybridization), and its overexpression was significantly correlated with poor prognosis and malignant progression among patients with glioma [32, 51, 99]. Furthermore, clinical database analyses indicated that the expression of LBX2-AS1 was remarkably higher in mesenchymal (MES) subtypes and recurrent glioma cases than in other types of glioma [32]. One bioinformatics study, based on the analysis of a weighted gene co-expression network, indicated that LBX2-AS1 was one of six differentially expressed, prognosis-related lncRNAs [51].

#### Thyroid cancer

Thyroid cancer (TC), is the most frequent endocrine malignancy, accounting for approximately 2.1% of all diagnosed cancer worldwide and the incidence of TC continues rise. Remarkably, 77% of these cases occurred in women [100, 101]. Standardized surgical treatment followed by radioactive iodine is effective for most patients with differentiated or medullary TC. Targeted therapy has prolonged progression-free survival, but the drugs are not curative and reserved for patients with progressive symptoms [102, 103]. Hence, it is significant to investigate the potential targeted strategies for the implications of TC patients. Recent study revealed that lncRNA LBX2-AS1 was obviously upregulated in TC tissue, compared with adjacent non-tumor thyroid tissue and high expression level of LBX2-AS1 was significantly correlated with clinical features of patients such as advanced tumor stages [27]. To verify its dysregulation, Li et al. performed in vitro experiments and discovered that LBX2-AS1 was significantly upregulated in TC cell lines (TPC1 and KTC-1), compared with normal human thyroid cell lines [27].

#### Other cancers

In addition to the specifically discussed tumor types, the dysregulation of LBX2-AS1 has also been reported in other cancer types. High LBX2-AS1 expression levels were detected in clinical tissue samples and cancer cell lines in studies focused on non-small cell lung cancer (NSCLC), esophageal squamous cell carcinoma (ESCC), and multiple myeloma (MM) [28, 53, 54]. Additionally, the high expression of LBX2-AS1 was significantly associated with advanced TNM staging, worse histological grades, and widespread lymph node metastasis, indicating a remarkable relationship between LBX2-AS1 expression and tumor progression. Kaplan–Meier survival analysis indicated that patients with high LBX2-AS1 expression levels had poor overall survival [26, 30]. Collectively, these investigations revealed abnormal overexpression profiles for LBX2-AS1 in multiple cancer types and identified significant correlations between LBX2-AS1 expression patterns and the clinicopathological features of cancer, suggesting that the lncRNA LBX2-AS1 likely plays important roles in the occurrence and development of human cancers.

### Xenograft model

To further explore the biological functions played by LBX2-AS1 in the progression of diverse cancers, in vivo tumor xenograft animal models were generated by investigators using GC, OC, glioma, HCC, TC, and MM cells, and the oncogenic effects of LBX2-AS1 on tumor growth (growth rate, volume, and weight) were observed. The volumes and weights of xenograft tumors were significantly smaller and lighter in groups in which LBX2-AS1 was inhibited by small hairpin RNA (shRNA; sh-LBX2-AS1) compared with groups treated with a negative control shRNA (shNC). In addition, the tumors in the shNC groups grew faster than those in the sh-LBX2-AS1 groups, indicating the powerful cancer-promoting effects of LBX2-AS1 [30, 47]. Knocking down LBX2-AS1 attenuated tumor development in vivo. Moreover, immunohistochemical (IHC) and hematoxylin-and-eosin staining assays showed that the numbers of Ki-67-positive and N-cadherin-positive cells and metastasizing nodules were significantly increased in the shNC groups compared with the sh-LBX2-AS1 groups [27, 32]. In lung metastasis model conducted by Fang et al., the upregulation of LBX2-AS1 was also observed to promote the number of tumor nodules [104]. Remarkably, in vivo experiments using patient-derived CRC xenografts showed that tumors with reduced LBX2-AS1 expression exhibited tumor growth suppression upon 5-FU treatment [44]. The tumor growth-promoting effects of LBX2-AS1 suggest that it functions as an oncogenic lncRNA during tumor formation.

## Biological functions of LBX2-AS1 and molecular mechanisms

In addition to LBX2-AS1 expression profiles in cancer tissues and cell lines, researchers have also explored the underlying mechanisms through which LBX2-AS1 mediates cancer-related biological functions and regulatory functions using molecular biology techniques, such as RNA immunoprecipitation (RIP) assay. Functionally, LBX2-AS1 modulates cancer-related pathophysiologic processes, such as cell growth and motility, by interacting with pivotal molecules in various cell signaling pathways. In the next section, we discuss the biological functions of LBX2-AS1, focusing on the underlying mechanisms that link multiple cascades. In addition, a summary showing the functions of LBX2-AS1, its upstream regulators, and downstream effectors in diverse cancer types is provided in Table 2.

**Table 2:** Biological functions and upstream–downstream regulation of LBX2-AS1 in various cancer cell lines

Cancer type	Cell lines	Upstream regulators	Targets	Downstream regulators/pathways	Tumor effect	Biological functions	References
GC	AGS, SGC-7901	LBX2	miR-219a-3p	FUS/LBX2	Oncogenic	Cell proliferation, apoptosis	[30]
GC	MGC803, BGC823, HGC27, SGC7901, GES1	NFIC	miR-491-5p	ZNF703	Oncogenic	Cell proliferation, apoptosis, invasion and migration	[39]
GC	MKN-45, BGC-823, SGC-7901, GES-1	–	miR-4766-5p	CXCL5	Oncogenic	Cell proliferation, invasion and migration	[40]
OC	SKOV3, OVCAR‐3, Caov‐3, ES‐2, HOSEPICs	–	miR‐455‐5p, miR-491-5p	E2F2	Oncogenic	Cell growth, apoptosis, clonogenicity, invasion and migration	[47]
OC	CaOV3, SKOV3, OVCAR3, OV90, HOSE		miR-4784	KDM5C	Oncogenic	Cell proliferation, apoptosis, colony formation, stemness, invasion and migration	[31]
CRC	HCT116, SW480	METTL3/IGF2BP1 m6A	miR-422a	AKT1	Oncogenic	Cell proliferation, invasion and migration	[44]
CRC	HT29, LoVo, SW620, HCT116, NCM460	–		CCND1, CDK3, CDK6, CDKN1A	Oncogenic	Cell proliferation	[29]
CRC	FHC, LoVo, SW620, HCT116, HT29	ELK-1	miR-491-5p	S100A11	Oncogenic	Cell proliferation, apoptosis, viability, colony formation, EMT, invasion and migration	[26]
CRC	SW480, HT29	–	PTPB1	KAT2A	Oncogenic	Cell proliferation, invasion and migration	[106]
CRC	SW480, HCT116, HCT-8, SW1116, HT29	–	miR-627-5p	RAC1/PI3K/AKT	Oncogenic	Cell growth, proliferation, apoptosis, EMT and metastasis	[104]
Glioma	U87, LN229, A172, T98G, U251, NHA, HUVECs, N3	SP-1	miR-491-5p	LIF-STAT3	Oncogenic	Cell proliferation, colony formation, viability, EMT, invasion and migration	[32]
Glioma	–	–		AKT/GSK3β	Oncogenic	Cell proliferation, viability, invasion and migration	[77]
AAA	VSMCs	LBX2	miR-4685-5p	LBX2	Oncogenic	Cell proliferation, apoptosis	[85]
HCC	L-O2, Huh7, Hep3B, SK-HEP-1	–	miR-384	IRS1	Oncogenic	Cell proliferation, apoptosis, invasion and migration	[52]
TC	HTori-3, TPC-1, KTC-1, and FTC-133	–	RARα	FSTL3	Oncogenic	Cell proliferation, viability, EMT, invasion and migration	[27]
MM	NCI-H929, U266, CD138 + plasmocytes	LBX2		LBX2 mRNA	Oncogenic	Cell viability, apoptosis, colony formation	[28]
NSCLC	A549, PC9, H1975, SPC-A1, H1299	–		Notch	Oncogenic	Cell proliferation, invasion and migration	[53]
ESCC	KYSE150, KYSE-410, KYSE450, EC109, EC9706, TE-13, HEEC	ZEB1	HNRNPC	ZEB1, ZEB2	Oncogenic	Cell migration, EMT	[54]

### Cell proliferation and apoptosis

**Fig. 1:**
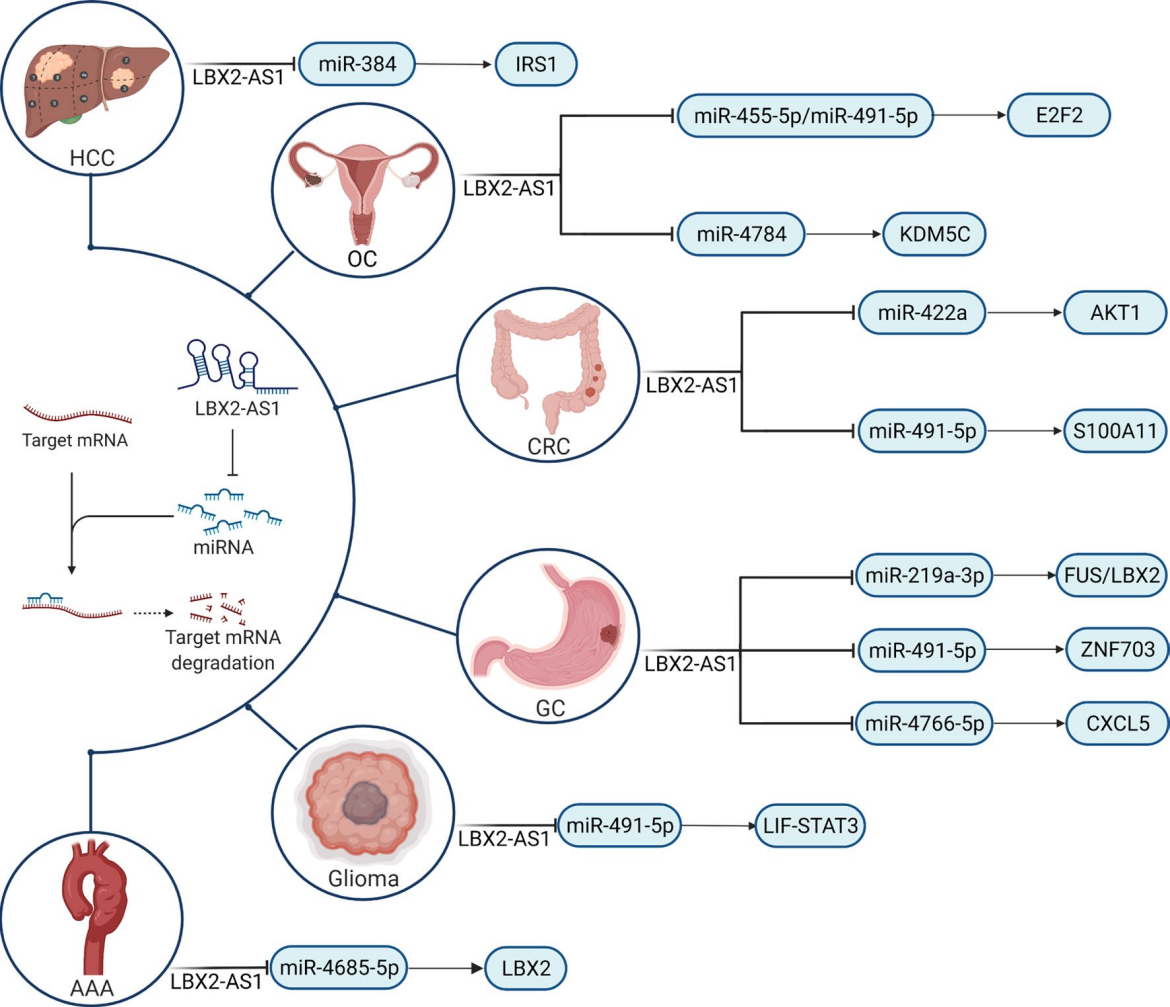
A comprehensive schematic representation of the lncRNA–miRNA–mRNA activities associated with LBX2-AS1 in diverse human cancers. MiRNAs are capable of interacting with the 3′-untranslated regions of protein-coding mRNAs, resulting in the degradation and translational repression of the targeted mRNA; however, the negative effects on mRNA translation can be counteracted by LBX2-AS1. In multiple cancers, LBX2-AS1 exerts CeRNA functions via various axis, modulating the expression of cancer-related signaling molecules/pathways. LBX2-AS1, ladybird homeobox 2-antisense RNA 1; lncRNA, long non-coding RNA; miRNAs, microRNAs; CeRNA, competing endogenous RNA

Broadly speaking, cancer can be described as abnormal and uncontrollable tissue growth with idiopathy and complicacy. Superficially, various types of tumors appear to be driven by different mechanisms, but crucial shared properties that define cancer hallmarks underlie the apparent diversity and heterogeneity of cancer [55, 56]. The unlimited proliferation of cancer cells, associated with repressed apoptosis, is one of the most significant cancer hallmarks, and LBX2-AS1 was universally demonstrated to exert crucial effects on cell proliferation and apoptosis across cancer types, including GC, MM, and CRC [57, 58]. One well-acknowledged mechanism through which lncRNA regulates cell functions is the molecular sponging of microRNAs (miRNAs) in the competing endogenous RNA (ceRNA) network (lncRNA-miRNA-mRNA) (Fig. 1) [18, 59–61].

MiRNAs feature complimentary sequences able to interact with the 3'-untranslated regions of protein-coding mRNAs, resulting in the degradation and translational repression of the targeted mRNA; however, some lncRNAs compete with these mRNAs to bind miRNAs, counteracting the negative effects on mRNA translation [62, 63]. Functional biological analyses have demonstrated that LBX2-AS1 fosters GC cell proliferation and inhibits apoptosis [30, 39]. Further investigations revealed that LBX2-AS1 acts as a ceRNA for fused in sarcoma (FUS), an oncogenic RNA-binding protein [64, 65, 105], by sponging miR-219a-3p [30]. Xu et al. demonstrated that LBX2-AS1 heightens the malignant phenotypes of GC cells through effects on the miR-491-5p-zinc finger protein 703 (ZNF703) axis [39]. Moreover, Peng et al. indicated that the miR-4766-5p-C-X-C motif chemokine ligand 5 (CXCL5) axis represents another significant pathway involved in the effects of LBX2-AS1 on GC cell growth [40]. In vitro experiments demonstrated that LBX2-AS1 positively regulates OC cell growth and colony formation via the miR-455-5p/miR-491-5p-E2F transcription factor 2 (E2F2) and miR4784-lysine demethylase 5C (KDM5C) axes [31, 47]. More recently, LBX2-AS1 has emerged as a ceRNA that accelerates the progression of CRC via the ETS-like protein (ELK1)-miR-422a-AKT1 and miR-491-5p-S100 calcium-binding protein A11 (S100A11) axes [26, 44]. In other cancer types, including glioma and HCC, LBX2-AS1 affects cell proliferation and apoptosis through the miR-491-5p-leukemia inhibitory factor (LIF)-signal transducer and activator of transcription 3 (STAT3) and miR-384-insulin receptor substrate 1 (IRS1) pathways, respectively [32, 52]. Collectively, LBX2-AS1-mediated ceRNA activity represents an essential contributor to neoplastic processes. Recent evidence indicates that the expression level of LBX2-AS1 is negatively correlated with that of cyclin-dependent kinase inhibitor 1A (CDKN1A) but positively correlated with that of cyclin-dependent kinase (CDK) 3, cyclin D1 (CCND1), and CDK6 [29]; however, the specific relationships between these signaling molecules remain to be further investigated.

### Cell invasion and migration

Cell invasion and migration are significant hallmark events, determined by alterations in cell shapes and behaviors as well as attachment to adjacent cells and the extracellular matrix. These events can transform malignancies from local growth into metastatic colonization, which associated with a high risk of mortality [66, 67]. Research into the mechanisms underlying metastasis has increased with the introduction of novel biological techniques and improved experimental tools, revealing the role of lncRNAs for this essential capacity of diverse cancer cells [58, 68, 69]. Mechanistically, LBX2-AS1 positively regulates cell invasion and migration in TC by interacting with retinoic acid receptor alpha (RARα) to activate follistatin-like protein 3 (FSTL3) [27]. In addition, Zhang et al. demonstrated that the interaction between LBX2-AS1 and RNA-binding protein heterogeneous nuclear ribonucleoprotein C (HNRNPC) strengthens the migration capacity and the progression of the epithelial-mesenchymal transition in ESCC cells by enhancing the stability of zinc finger E-box binding homeobox (ZEB) 1/2 mRNA, which has been implicated in the reinforcement of cell invasion capabilities [70–72]. Further investigations showed that ZEB1 but not ZEB2 activates LBX2-AS1 transcription, forming a positive feedback loop [54]. The dysregulation of signal transduction pathways, which represent essential regulatory mechanisms for intracellular or extracellular stimuli associated with diverse biological processes, including cell invasion and migration, has been broadly implicated in cancer occurrence and metastasis [73–76]. Over the past decades, dual luciferase reporter and gain- and loss-of-function assays have revealed that LBX2-AS1 is capable of regulating the AKT-glycogen synthase kinase-3 beta (GSK3β) and Notch pathways [53, 77], which play crucial functions in enhancing cancer cell motility and contribute to cancer metastasis [78–81].

### Upstream regulation of LBX2-AS1

**Fig. 2:**
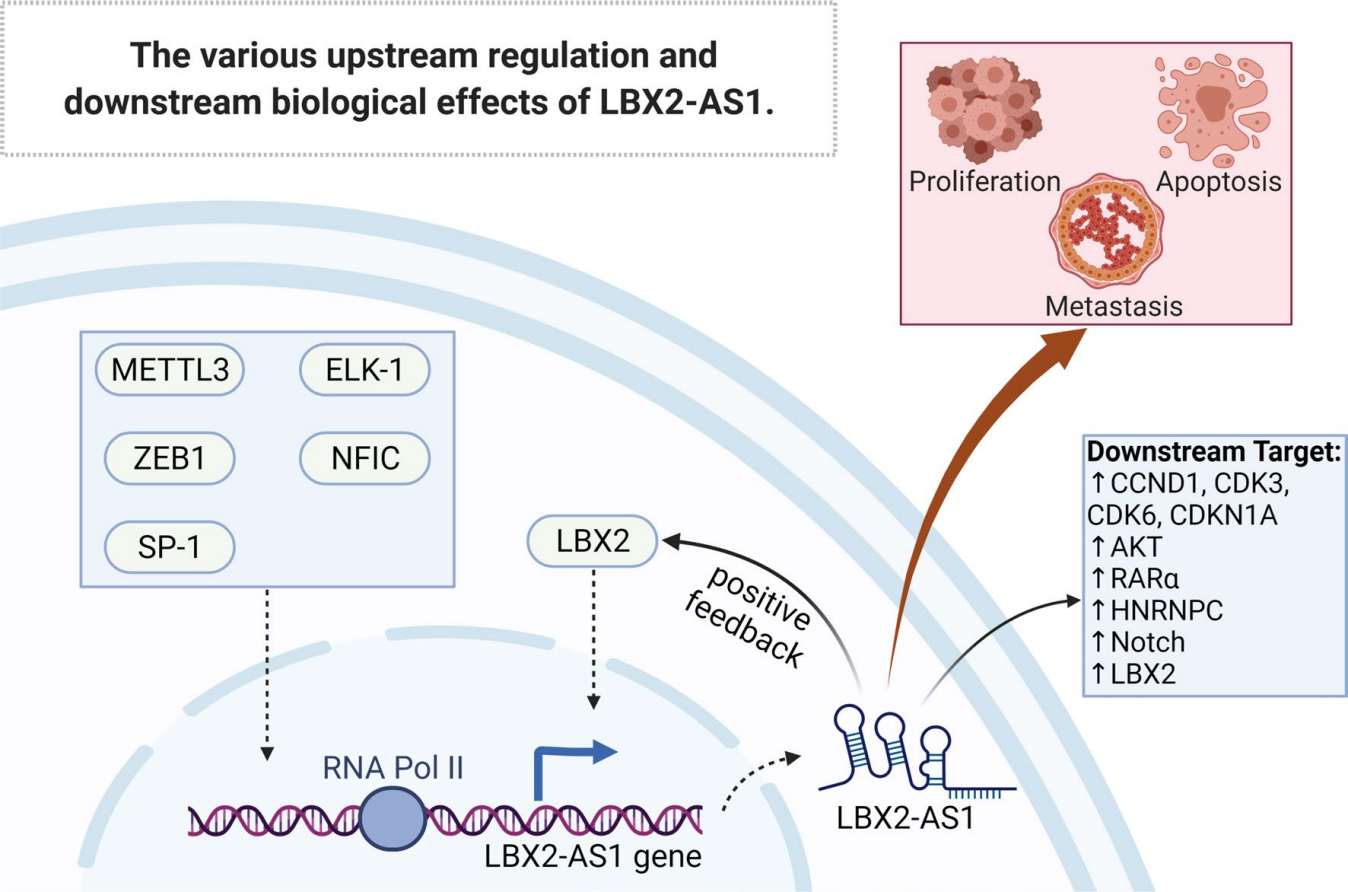
The various upstream regulatory mechanisms and downstream biological effects mediated by LBX2-AS1. The expression of LBX2-AS1 is regulated by multiple transcription factors, such as LBX2 and ZEB1, and LBX2 might also be regulated by LBX2-AS1, forming a positive feedback loop. METTL3-mediated RNA methylation is also involved in LBX2-AS1 regulation. Downstream effects of LBX2-AS1 include essential cancer-related events, such as sustained proliferation and hypoactive apoptosis. LBX2-AS1, ladybird homeobox 2-antisense RNA 1; LBX2, ladybird homeobox 2; ZEB1, zinc finger E-box binding homeobox (ZEB) 1; METTL13, methyltransferase-like 3

The transcription factor (TF) LBX2 is a significant member of the homeobox gene family that plays crucial roles in biological growth processes. Lu et al. demonstrated that LBX2 positively modulates gene transcription by relieving the suppression of Wnt/β-catenin signaling pathway activation [82–84]. Existing evidence indicates that LBX2 is crucially involved in the upstream regulation of LBX2-AS1. More intriguingly, LBX2-AS1 enhances LBX2 mRNA stability via FUS, and LBX2, in turn, enhances the expression of LBX2-AS1, revealing a positive feedback loop mediated by LBX2-AS1. Another study also described a positive feedback loop involving LBX2-AS1, miR-4685-5p, and LBX2 in abdominal aortic aneurysms (AAA) [28, 30, 85]. Two additional TFs, nuclear factor I C (NFIC) and SP-1, also enhance the transcription of LBX2-AS1 [32, 39]. In addition, Ma et al. revealed that the binding of LBX2-AS1 with insulin-like growth factor 2 mRNA-binding protein 1 (IGF2BP1) resulted in the m6a hyper-methylation of LBX2-AS1 by methyltransferase-like 3 (METTL3), stabilizing and upregulating LBX2-AS1 [44]. ELK1 has recently been identified as another upstream regulator of LBX2-AS1, capable of binding with two conserved sequence motifs in the LBX2-AS1 promoter to drive LBX2-AS1 transcription [26]. The various up-stream regulators and biological effects are signed in Fig. 2.

## Clinical utility of LBX2-AS1 in oncology

Biomarkers describe biological substances or active agents that can be objectively detected and measured in tissue samples, serum, or exosomes and include diverse proteins, RNA transcripts, DNA, and metabolites capable of providing robust information regarding the particular stages, pathological processes, or therapeutic reactions of diseases. Researchers have attempted to identify cancer biomarkers for use in early disease diagnosis, accurate tumor classification, prognosis prediction, and therapeutic response evaluation to provide cancer patients with timely interventions and more integrative and productive clinical disease management [86–90]. The seeking for valid and effective cancer biomarkers for use in human cancers has been ongoing for several decades, with considerable progress made possible by advances in available experimental tools and biologic technology. In recent years, several biomarkers have emerged as valuable indicators for diverse cancers. For example, human epidermal growth factor receptor 2 (HER2) is a positive biomarker for the response to trastuzumab in patients with GC, and the amplification of HER2 serves as a molecular biomarker for patients with breast cancer (BC) [91–93]. Prostate-specific antigen (PSA) is a universally acknowledged biomarker for prostate cancer (PC) has been commonly used to screen and monitor PC progression for long, but it has come into controversy these years for its intrinsic limitation in terms of accuracy and specificity [94–96]. Currently available biomarkers are not always detectable, and cancers can be associated with a broad array of etiopathogenetic mechanisms, suggesting the need for additional cancer biomarkers to be identified.

In the previous section, we discuss the upregulation of LBX2-AS1 expression in various cancers. Due to the high correlation between LBX2-AS1 overexpression and important clinicopathological features in cancer patients, including tumor stage, metastasis, and prognosis, LBX2-AS1 may represent a lncRNA with great clinical value, attracting the attention of researchers who have attempted to validate its potential role as a novel biomarker. The results of independent research studies revealed that LBX2-AS1 was consistently upregulated in CRC tissues (401 CRC samples in total), particularly among patients with advanced tumor stages and poor prognosis [26, 29, 44]. Multivariate Cox regression and receiver operating characteristic curve analyses [area under the curve (AUC) = 0.7595, *P* < 0.001] indicated that LBX2-AS1 represented a promising independent prognostic factor and diagnostic biomarker for patients with CRC [26]. Liang et al. constructed a risk-scoring model consisting of six lncRNAs, including LBX2-AS1, which were selected from bioinformatics analysis. The prognostic value of this scoring model was validated using two separate datasets [44]. The promising diagnostic value of circulating LBX2-AS1 was also verified in another study of 60 patients with MM [28]. These findings indicated that LBX2-AS1 might represent a favorable indicator for early cancer diagnosis and prognosis prediction. Overwhelming evidence has indicated the oncogenic roles of LBX2-AS1 in the occurrence and development of diverse cancers. Encouragingly, the extensive interacting networks through which LBX2-AS1 exerts its regulatory functions and enhances the malignancy of various tumors have been thoroughly investigated, making LBX2-AS1-targeted therapy an intriguing possibility. As we discussed previously, LBX2-AS1 has powerful effects on cancer cell proliferation, invasion, and apoptosis. Additionally, in vivo xenograft experiments show that LBX2-AS1 expression levels are negatively correlated with the 5-FU treatment response, which has been verified in clinical investigations [44]. Additional well-designed clinical trials remain necessary to examine the practical applications of LBX2-AS1-targeted therapy.

## Conclusion

LBX2-AS1 is a remarkable lncRNA that has been demonstrated to play significant roles in human cancer development over the past decades. We provide a comprehensive summary of research showing that LBX2-AS1 is critically upregulated in diverse cancer samples and cancer cell lines (GC, OC, CRC, HCC, TC, etc.). More meaningfully, the overexpression of LBX2-AS1 is highly associated with the clinical characteristics of patients, such as tumor stage, lymph node metastasis, and overall survival rate, indicating that LBX2-AS1 has great clinical value as a novel cancer biomarker. The powerful tumor growth-promoting effects of LBX2-AS1 were verified in vivo using tumor xenograft models. Overwhelming research supports the oncogenic functions of LBX2-AS1 in cancer initiation and progression. By applying experimental tools and new technologies, such as RIP and gain- and loss-of-function assays, researchers have revealed that LBX2-AS1 is capable of modulating tumor-related biological processes, including cell proliferation, apoptosis, and migration, via complex regulatory networks involving ceRNA activity, protein–lncRNA interactions, and positive feedback loops that regulate LBX2-AS1 expression, indicating the potential benefits of therapeutic strategies that target this lncRNA.

Despite a general consensus regarding the oncogenic functions and clinical applications of LBX2-AS1, some challenges and controversies remain to be addressed. For example, whether LBX2-AS1 is sufficiently stable and measurable to serve as a consistent biomarker and therapeutic target remains unclear. Overall, the significant roles played by LBX2-AS1 in human cancer development and progression indicate its tremendous potential for use in oncology. An improved understanding of the functional mechanisms underlying these roles is likely to continue revealing promising management strategies for cancer patients.

## Abbreviations

AAA: Abdominal aortic aneurysms
AUC: Area under the curve
BC: Breast cancer
CDKN1A: Cyclin-dependent kinase inhibitor 1A
CDK: Cyclin-dependent kinase
ceRNA: Competing endogenous RNA
CXCL5: Chemokine ligand 5
CRC: Colorectal cancer
CTCL: Cutaneous T-cell lymphomas
E2F2: E2F transcription factor 2
ELK1: ETS-like protein
ESCC: Esophageal squamous cell carcinoma
FISH: Fluorescence in situ hybridization
FSTL3: Follistatin-like protein 3
GC: Gastric cancer
GES-1: Gastric mucosa epithelial cells
GSK3β: Glycogen synthase kinase-3 beta
HCC: Hepatocellular carcinoma
HER2: Human epidermal growth factor receptor 2
hTERT: Human telomerase reverse transcriptase
hTR: Human telomerase RNA
HNRNPC: Heterogeneous nuclear ribonucleoprotein C
IGF2BP1: Insulin-like growth factor 2 mRNA-binding protein 1
IHC: Immunohistochemical
IRS1: Insulin receptor substrate 1
KDM5C: Lysine demethylase 5C
LBX2: Ladybird homeobox 2
LBX2-AS1: Ladybird homeobox 2-antisense RNA 1
LIF: Leukemia inhibitory factor
lncRNAs: Long non-coding RNAs
MES: Mesenchymal
miRNAs: MicroRNAs
MM: Multiple myeloma
NFIC: Nuclear factor I C
OC: Ovarian cancer
PCGEM1: Prostate cancer gene expression marker 1
PSA: Prostate-specific antigen
RARα: Retinoic acid receptor alpha
RIP: RNA immunoprecipitation
shRNA: Small hairpin RNA
shNC: Negative control shRNA
STAT3: Signal transducer and activator of transcription 3
S100A11: S100 calcium-binding protein A11
TC: Thyroid cancer
TERRA: Telomeric repeat-containing RNA
TF: Transcription factor
TNM: Tumor–node–metastasis
ZEB: Zinc finger E-box binding homeobox
5-FU: 5-Fluorouracil

## References

[1] Bhan A, Soleimani M, Mandal SS (2017). Long noncoding RNA and cancer: a new paradigm. Cancer Res.

[2] Sung H, Ferlay J, Siegel RL, Laversanne M, Soerjomataram I, Jemal A (2021). Global Cancer Statistics 2020: GLOBOCAN estimates of incidence and mortality worldwide for 36 cancers in 185 countries. CA Cancer J Clin.

[3] Gilbertson RJ (2011). Mapping cancer origins. Cell.

[4] Mun EJ, Babiker HM, Weinberg U, Kirson ED, Von Hoff DD (2018). Tumor-treating fields: a fourth modality in cancer treatment. Clin Cancer Res.

[5] Wang JJ, Lei KF, Han F (2018). Tumor microenvironment: recent advances in various cancer treatments. Eur Rev Med Pharmacol Sci.

[6] Boshuizen J, Peeper DS (2020). Rational cancer treatment combinations: an urgent clinical need. Mol Cell.

[7] Tsimberidou AM, Fountzilas E, Nikanjam M, Kurzrock R (2020). Review of precision cancer medicine: evolution of the treatment paradigm. Cancer Treat Rev.

[8] Von Hoff DD, Stephenson JJ, Rosen P, Loesch DM, Borad MJ, Anthony S (2010). Pilot study using molecular profiling of patients' tumors to find potential targets and select treatments for their refractory cancers. J Clin Oncol.

[9] Le Tourneau C, Delord JP, Gonçalves A, Gavoille C, Dubot C, Isambert N (2015). Molecularly targeted therapy based on tumour molecular profiling versus conventional therapy for advanced cancer (SHIVA): a multicentre, open-label, proof-of-concept, randomised, controlled phase 2 trial. Lancet Oncol.

[10] Schwaederle M, Parker BA, Schwab RB, Daniels GA, Piccioni DE, Kesari S (2016). Precision oncology: the UC San Diego Moores Cancer Center PREDICT experience. Mol Cancer Ther.

[11] Kurzrock R, Giles FJ (2015). Precision oncology for patients with advanced cancer: the challenges of malignant snowflakes. Cell Cycle.

[12] Dillies MA, Rau A, Aubert J, Hennequet-Antier C, Jeanmougin M, Servant N (2013). A comprehensive evaluation of normalization methods for Illumina high-throughput RNA sequencing data analysis. Brief Bioinform.

[13] Pareek CS, Smoczynski R, Tretyn A (2011). Sequencing technologies and genome sequencing. J Appl Genet.

[14] Djebali S, Davis CA, Merkel A, Dobin A, Lassmann T, Mortazavi A (2012). Landscape of transcription in human cells. Nature.

[15] Atianand MK, Hu W, Satpathy AT, Shen Y, Ricci EP, Alvarez-Dominguez JR (2016). A long noncoding RNA lincRNA-EPS acts as a transcriptional brake to restrain inflammation. Cell.

[16] Harrow J, Frankish A, Gonzalez JM, Tapanari E, Diekhans M, Kokocinski F (2012). GENCODE: the reference human genome annotation for The ENCODE Project. Genome Res.

[17] Zhao Y, Li H, Fang S, Kang Y, Wu W, Hao Y (2016). NONCODE 2016: an informative and valuable data source of long non-coding RNAs. Nucleic Acids Res.

[18] Schmitz SU, Grote P, Herrmann BG (2016). Mechanisms of long noncoding RNA function in development and disease. Cell Mol Life Sci.

[19] Ransohoff JD, Wei Y, Khavari PA (2018). The functions and unique features of long intergenic non-coding RNA. Nat Rev Mol Cell Biol.

[20] Sanchez Calle A, Kawamura Y, Yamamoto Y, Takeshita F, Ochiya T (2018). Emerging roles of long non-coding RNA in cancer. Cancer Sci.

[21] Peng WX, Koirala P, Mo YY (2017). LncRNA-mediated regulation of cell signaling in cancer. Oncogene.

[22] Tan YT, Lin JF, Li T, Li JJ, Xu RH, Ju HQ (2021). LncRNA-mediated posttranslational modifications and reprogramming of energy metabolism in cancer. Cancer Commun (Lond)..

[23] Li J, Meng H, Bai Y, Wang K (2016). Regulation of lncRNA and its role in cancer metastasis. Oncol Res.

[24] Zhang QJ, Li DZ, Lin BY, Geng L, Yang Z, Zheng SS (2022). SNHG16 promotes hepatocellular carcinoma development via activating ECM receptor interaction pathway. Hepatobiliary Pancreat Dis Int.

[25] Liu SL, Chen MH, Wang XB, You RK, An XW, Zhao Q (2021). LncRNA PCGEM1 contributes to malignant behaviors of glioma by regulating miR-539–5p/CDK6 axis. Aging (Albany NY)..

[26] Ma G, Dai W, Zhang J, Li Q, Gu B, Song Y (2021). ELK1-mediated upregulation of lncRNA LBX2-AS1 facilitates cell proliferation and invasion via regulating miR-491-5p/S100A11 axis in colorectal cancer. Int J Mol Med.

[27] Li J, Shen J, Qin L, Lu D, Ding E (2021). LBX2-AS1 activates FSTL3 by binding to transcription factor RARα to foster proliferation, migration, and invasion of thyroid cancer. Front Genet.

[28] Jia H, Liu Y, Lv S, Qiao R, Zhang X, Niu F (2021). LBX2-AS1 as a novel diagnostic biomarker and therapeutic target facilitates multiple myeloma progression by enhancing mRNA stability of LBX2. Front Mol Biosci.

[29] Li Q, Xie H, Jin Z, Huang J, Wang S, Zhang Z (2021). Overexpression of long noncoding RNA LBX2-AS1 promotes the proliferation of colorectal cancer. Technol Cancer Res Treat.

[30] Yang Z, Dong X, Pu M, Yang H, Chang W, Ji F (2020). LBX2-AS1/miR-219a-2–3p/FUS/LBX2 positive feedback loop contributes to the proliferation of gastric cancer. Gastric Cancer.

[31] Gu H, Lin R, Zheng F, Zhang Q (2021). ELK1 activated-long noncoding RNA LBX2-AS1 aggravates the progression of ovarian cancer through targeting miR-4784/KDM5C axis. J Mol Histol.

[32] Li W, Soufiany I, Lyu X, Lu C, Wei Y, Shi Z (2021). SP1-upregulated LBX2-AS1 promotes the progression of glioma by targeting the miR-491–5p/LIF axis. J Cancer.

[33] Jin SH, Zhou RH, Guan XY, Zhou JG, Liu JG (2020). Identification of novel key lncRNAs involved in periodontitis by weighted gene co-expression network analysis. J Periodontal Res.

[34] Thrift AP, El-Serag HB (2020). Burden of gastric cancer. Clin Gastroenterol Hepatol.

[35] Karimi P, Islami F, Anandasabapathy S, Freedman ND, Kamangar F (2014). Gastric cancer: descriptive epidemiology, risk factors, screening, and prevention. Cancer Epidemiol Biomarkers Prev.

[36] Zong L, Abe M, Seto Y, Ji J (2016). The challenge of screening for early gastric cancer in China. Lancet.

[37] Sexton RE, Al Hallak MN, Diab M, Azmi AS (2020). Gastric cancer: a comprehensive review of current and future treatment strategies. Cancer Metastasis Rev.

[38] Johnston FM, Beckman M (2019). Updates on management of gastric cancer. Curr Oncol Rep.

[39] Xu G, Zhang Y, Li N, Wu Y, Zhang J, Xu R (2020). LBX2-AS1 up-regulated by NFIC boosts cell proliferation, migration and invasion in gastric cancer through targeting miR-491–5p/ZNF703. Cancer Cell Int.

[40] Peng L, Chen Z, Wang G, Tian S, Kong S, Xu T (2020). Long noncoding RNA LBX2-AS1-modulated miR-4766–5p regulates gastric cancer development through targeting CXCL5. Cancer Cell Int.

[41] Center MM, Jemal A, Smith RA, Ward E (2009). Worldwide variations in colorectal cancer. CA Cancer J Clin.

[42] Bosetti C, Levi F, Rosato V, Bertuccio P, Lucchini F, Negri E (2011). Recent trends in colorectal cancer mortality in Europe. Int J Cancer.

[43] Guo P, Huang ZL, Yu P, Li K (2012). Trends in cancer mortality in China: an update. Ann Oncol.

[44] Ma YN, Hong YG, Yu GY, Jiang SY, Zhao BL, Guo A (2021). LncRNA LBX2-AS1 promotes colorectal cancer progression and 5-fluorouracil resistance. Cancer Cell Int.

[45] Webb PM, Jordan SJ (2017). Epidemiology of epithelial ovarian cancer. Best Pract Res Clin Obstet Gynaecol.

[46] Moufarrij S, Dandapani M, Arthofer E, Gomez S, Srivastava A, Lopez-Acevedo M (2019). Epigenetic therapy for ovarian cancer: promise and progress. Clin Epigenet.

[47] Cao J, Wang H, Liu G, Tang R, Ding Y, Xu P (2021). LBX2-AS1 promotes ovarian cancer progression by facilitating E2F2 gene expression via miR-455–5p and miR-491–5p sponging. J Cell Mol Med.

[48] Meng C, Zhou JQ, Liao YS (2020). Autophagy-related long non-coding RNA signature for ovarian cancer. J Int Med Res.

[49] Ostrom QT, Gittleman H, Farah P, Ondracek A, Chen Y, Wolinsky Y (2013). CBTRUS statistical report: primary brain and central nervous system tumors diagnosed in the United States in 2006–2010. Neuro Oncol.

[50] Ostrom QT, Bauchet L, Davis FG, Deltour I, Fisher JL, Langer CE (2014). The epidemiology of glioma in adults: a “state of the science” review. Neuro Oncol.

[51] Liang R, Zhi Y, Zheng G, Zhang B, Zhu H, Wang M (2019). Analysis of long non-coding RNAs in glioblastoma for prognosis prediction using weighted gene co-expression network analysis, Cox regression, and L1-LASSO penalization. Onco Targets Ther.

[52] Wang Y, Zhao Y, Zhang X, Zhang A, Ma J (2020). Long noncoding RNA LBX2-AS1 drives the progression of hepatocellular carcinoma by sponging microRNA-384 and thereby positively regulating IRS1 expression. Pathol Res Pract.

[53] Tang LX, Su SF, Wan Q, He P, Xhang Y, Cheng XM (2019). Novel long non-coding RNA LBX2-AS1 indicates poor prognosis and promotes cell proliferation and metastasis through Notch signaling in non-small cell lung cancer. Eur Rev Med Pharmacol Sci.

[54] Zhang Y, Chen W, Pan T, Wang H, Zhang Y, Li C (2019). LBX2-AS1 is activated by ZEB1 and promotes the development of esophageal squamous cell carcinoma by interacting with HNRNPC to enhance the stability of ZEB1 and ZEB2 mRNAs. Biochem Biophys Res Commun.

[55] Hanahan D, Weinberg RA (2000). The hallmarks of cancer. Cell.

[56] Macheret M, Halazonetis TD (2015). DNA replication stress as a hallmark of cancer. Annu Rev Pathol.

[57] Evan GI, Vousden KH (2001). Proliferation, cell cycle and apoptosis in cancer. Nature.

[58] Hanahan D, Weinberg RA (2011). Hallmarks of cancer: the next generation. Cell.

[59] Zhang L, Li C, Su X (2020). Emerging impact of the long noncoding RNA MIR22HG on proliferation and apoptosis in multiple human cancers. J Exp Clin Cancer Res.

[60] Rashid F, Shah A, Shan G (2016). Long non-coding RNAs in the cytoplasm. Genomics Proteomics Bioinform.

[61] Wei C, Luo T, Zou S, Zhou X, Shen W, Ji X (2017). Differentially expressed lncRNAs and miRNAs with associated ceRNA networks in aged mice with postoperative cognitive dysfunction. Oncotarget.

[62] Bartel DP, Chen CZ (2004). Micromanagers of gene expression: the potentially widespread influence of metazoan microRNAs. Nat Rev Genet.

[63] Weidle UH, Birzele F, Kollmorgen G, Rüger R (2017). Long non-coding RNAs and their role in metastasis. Cancer Genomics Proteomics.

[64] Jia W, Kim SH, Scalf MA, Tonzi P, Millikin RJ, Guns WM (2021). Fused in sarcoma regulates DNA replication timing and kinetics. J Biol Chem.

[65] Wu Y, Poulos RC, Reddel RR (2020). Role of POT1 in human cancer. Cancers (Basel)..

[66] Friedl P, Alexander S (2011). Cancer invasion and the microenvironment: plasticity and reciprocity. Cell.

[67] Lamouille S, Xu J, Derynck R (2014). Molecular mechanisms of epithelial–mesenchymal transition. Nat Rev Mol Cell Biol.

[68] Sanz-Moreno V, Marshall CJ (2010). The plasticity of cytoskeletal dynamics underlying neoplastic cell migration. Curr Opin Cell Biol.

[69] Talmadge JE, Fidler IJ (2010). AACR centennial series: the biology of cancer metastasis: historical perspective. Cancer Res.

[70] Soleymani L, Zarrabi A, Hashemi F, Hashemi F, Zabolian A, Banihashemi SM (2021). Role of ZEB family members in proliferation, metastasis, and chemoresistance of prostate cancer cells: revealing signaling networks. Curr Cancer Drug Targets.

[71] Browne G, Sayan AE, Tulchinsky E (2010). ZEB proteins link cell motility with cell cycle control and cell survival in cancer. Cell Cycle.

[72] Chen B, Chen B, Zhu Z, Ye W, Zeng J, Liu G (2019). Prognostic value of ZEB-1 in solid tumors: a meta-analysis. BMC Cancer.

[73] Siebel C, Lendahl U (2017). Notch signaling in development, tissue homeostasis, and disease. Physiol Rev.

[74] Steinhart Z, Angers S (2018). Wnt signaling in development and tissue homeostasis. Development.

[75] Vander Ark A, Cao J, Li X (2018). TGF-β receptors: In and beyond TGF-β signaling. Cell Signal.

[76] Sever R, Brugge JS (2015). Signal transduction in cancer. Cold Spring Harb Perspect Med.

[77] Wen H, Li Z, Song S, Xu L, Tong X, Yan H (2021). Silencing of lncRNA LBX2-AS1 suppresses glioma cell proliferation and metastasis through the Akt/GSK3β pathway in vitro. Acta Biochim Biophys Sin (Shanghai).

[78] Jiang H, Zhou Z, Jin S, Xu K, Zhang H, Xu J (2018). PRMT9 promotes hepatocellular carcinoma invasion and metastasis via activating PI3K/Akt/GSK-3β/Snail signaling. Cancer Sci.

[79] Xu W, Yang Z, Lu N (2015). A new role for the PI3K/Akt signaling pathway in the epithelial-mesenchymal transition. Cell Adh Migr.

[80] Zanotti S, Canalis E (2016). Notch signaling and the skeleton. Endocr Rev.

[81] Edwards A, Brennan K (2021). Notch Signalling in Breast Development and Cancer. Front Cell Dev Biol..

[82] Lu FI, Sun YH, Wei CY, Thisse C, Thisse B (2014). Tissue-specific derepression of TCF/LEF controls the activity of the Wnt/β-catenin pathway. Nat Commun.

[83] Chen F, Collin GB, Liu KC, Beier DR, Eccles M, Nishina PM (2001). Characterization of the murine Lbx2 promoter, identification of the human homologue, and evaluation as a candidate for Alström syndrome. Genomics.

[84] Moisan V, Robert NM, Tremblay JJ (2010). Expression of ladybird-like homeobox 2 (LBX2) during ovarian development and folliculogenesis in the mouse. J Mol Histol.

[85] Li H, Zhang H, Wang G, Chen Z, Pan Y (2020). LncRNA LBX2-AS1 facilitates abdominal aortic aneurysm through miR-4685–5p/LBX2 feedback loop. Biomed Pharmacother.

[86] Wu L, Qu X (2015). Cancer biomarker detection: recent achievements and challenges. Chem Soc Rev.

[87] Prensner JR, Rubin MA, Wei JT, Chinnaiyan AM. Beyond PSA: the next generation of prostate cancer biomarkers. Sci Transl Med. 2012;4(127):127rv3. 10.1126/scitranslmed.3003180 PMC3799996 22461644

[88] Ilyin SE, Belkowski SM, Plata-Salamán CR (2004). Biomarker discovery and validation: technologies and integrative approaches. Trends Biotechnol.

[89] Golubnitschaja O, Flammer J (2007). What are the biomarkers for glaucoma?. Surv Ophthalmol.

[90] Lindner JR, Link J (2018). Molecular imaging in drug discovery and development. Circ Cardiovasc Imaging.

[91] Oakman C, Santarpia L, Di Leo A (2010). Breast cancer assessment tools and optimizing adjuvant therapy. Nat Rev Clin Oncol.

[92] Nakamura Y, Kawazoe A, Lordick F, Janjigian YY, Shitara K (2021). Biomarker-targeted therapies for advanced-stage gastric and gastro-oesophageal junction cancers: an emerging paradigm. Nat Rev Clin Oncol.

[93] Jordan NV, Bardia A, Wittner BS, Benes C, Ligorio M, Zheng Y (2016). HER2 expression identifies dynamic functional states within circulating breast cancer cells. Nature.

[94] Lilja H, Ulmert D, Vickers AJ (2008). Prostate-specific antigen and prostate cancer: prediction, detection and monitoring. Nat Rev Cancer.

[95] Stenman UH, Leinonen J, Zhang WM, Finne P (1999). Prostate-specific antigen. Semin Cancer Biol.

[96] Saini S (2016). PSA and beyond: alternative prostate cancer biomarkers. Cell Oncol (Dordr).

[97] El Hajj J, Nguyen E, Liu Q, Bouyer C, Adriaenssens E, Hilal G (2018). Telomerase regulation by the long non-coding RNA H19 in human acute promyelocytic leukemia cells. Mol Cancer.

[98] Chebly A, Ropio J, Baldasseroni L, Prochazkova-Carlotti M, Idrissi Y, Ferrer J (2022). Telomeric repeat-containing RNA (TERRA): a review of the literature and first assessment in cutaneous T-cell lymphomas. Genes (Basel)..

[99] Wu F, Zhao Z, Chai R, Liu Y, Wang K, Wang Z (2018). Expression profile analysis of antisense long non-coding RNA identifies WDFY3-AS2 as a prognostic biomarker in diffuse glioma. Cancer Cell Int.

[100] Kitahara CM, Sosa JA (2016). The changing incidence of thyroid cancer. Nat Rev Endocrinol.

[101] Cabanillas ME, McFadden DG, Durante C (2016). Thyroid cancer. Lancet.

[102] Filetti S, Durante C, Hartl D, Leboulleux S, Locati LD, Newbold K (2019). Thyroid cancer: ESMO Clinical Practice Guidelines for diagnosis, treatment and follow-up†. Ann Oncol.

[103] Schneider DF, Chen H (2013). New developments in the diagnosis and treatment of thyroid cancer. CA Cancer J Clin.

[104] Fang J, Yang J, Chen H, Sun W, Xiang L, Feng J (2022). Long non-coding RNA LBX2-AS1 predicts poor survival of colon cancer patients and promotes its progression via regulating miR-627–5p/RAC1/PI3K/AKT pathway. Hum Cell.

[105] He Z, Ruan X, Liu X, Zheng J, Liu Y, Liu L (2019). FUS/circ_002136/miR-138–5p/SOX13 feedback loop regulates angiogenesis in Glioma. J Exp Clin Cancer Res.

[106] Zhao A, Wang Y, Lin F, Bai K, Gu C (2022). Long noncoding RNA LBX2-AS1 promotes colorectal cancer progression via binding with PTBP1 and stabilizing KAT2A expression. J Biochem Mol Toxicol.

